# The Therapeutic Efficacy of *Punica granatum* and Its Bioactive Constituents with Special Reference to Photodynamic Therapy

**DOI:** 10.3390/plants11212820

**Published:** 2022-10-24

**Authors:** Nosipho Thembekile Fakudze, Eric Chekwube Aniogo, Blassan P. George, Heidi Abrahamse

**Affiliations:** Laser Research Centre, Faculty of Health Sciences, University of Johannesburg, P.O. Box 17011, Doornfontein 2028, South Africa

**Keywords:** *P. granatum*, anticancer, antioxidant, anti-osteoarthritis

## Abstract

*Punica granatum (P. granatum)* is a fruit-bearing tree from the Punicaceae family, indigenous to Iran. This plant has healing qualities that have drawn the interest of the medical community as an alternative treatment for malignancies and non-malignancies. Its healing quality is due to the phytochemicals present in the plant. These include ellagic acid, punicic acid, phenols, and flavonoids. In traditional medicine, *P. granatum* has been used in treating diseases such as dysentery, bleeding disorders, leprosy, and burns. This review explores the effects of the phytochemical constituents of *P. granatum* on photodynamic therapy for cancer, chronic inflammation, osteoarthritis, and viral infections. Its antioxidant and antitumor effects play a role in reduced free radical damage and cancer cell proliferation. It was concluded that *P. granatum* has been used for many disease conditions for a better therapeutic outcome. This paper will give visibility to more studies and expand the knowledge on the potential use of *P. granatum* in photodynamic cancer treatment.

## 1. Introduction

**Figure 1:**
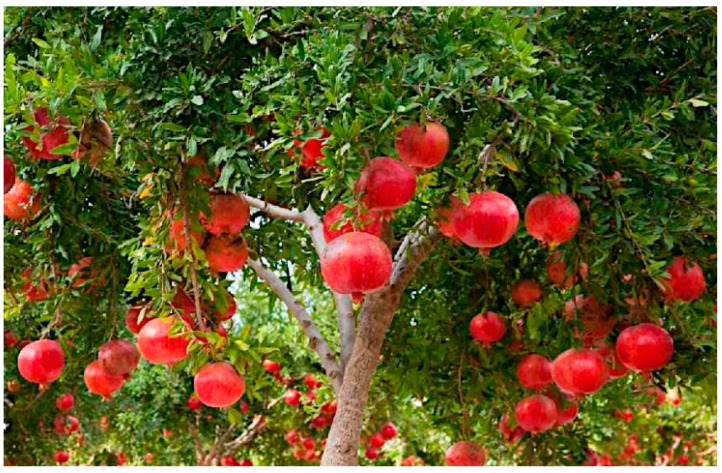
A picture of the *P. granatum* plant.

*Punica granatum* (pomegranate) is a small shrub or tree that belongs to the family Punicaceae, depicted in Figure 1 [1,2]. This tree grows to about 3 to 5 m with shiny, spear-shaped leaves, big white, red or multi-colored flowers, and fruits [3]. It is indigenous to Iran and Afghanistan but cultivated in Africa, Europe, and South and North America [4]. The history of the pomegranate shows that it was widely used as folk medicine in countries like Greece and Russia. Doctors described its juice as the medical treatment for various illnesses in Greece, including inflammation, dysentery, diarrhea, persistent coughs, and intestinal worms. At the same time, in the Georgian Republic of Russia, it was believed to inhibit inert hemorrhages, diarrhea, chronic mucous discharges, and night sweats [5].

The plant parts are all utilized in traditional medicine, especially in the Ayurvedic system [3]. The early conventional medicine systems used *P. granatum* in their herbal (drug) formulation for the Unani system, Ayurveda system, and traditional Chinese medicine in the treatment of diseases. Traditional medicine falls into the category of naturopathic medicine. It forms part of Western medicine in homeopathy and is in its infancy [6]. *P. granatum* comprises phytochemicals that assist in anticancer and antioxidant effects on acute or chronic conditions [3,7,8]. These phytochemicals are responsible for specific mechanisms of action that result in the diminished effect or elimination of cancer cells. The primary classes of phytochemicals are ellagic acid (antioxidant and anticancer properties), flavonoids (antiproliferation properties), and anthocyanins (antioxidant, antiviral, anti-inflammatory) [9,10,11].

The medical community has directed its attention to *P. granatum* in cancer therapy, treating diabetes, and chronic inflammation [12,13]. The phytochemicals in *P. granatum* have been used in many in vivo and in vitro studies. These include the treatment of cancers, such as skin, breast, prostate, oral, colon, etc., with positive therapeutic outcomes [14,15]. Phytochemicals are also used in photodynamic therapy for cancer [16,17,18,19]. These phytochemicals are used as photosensitizers and include riboflavin, punicalagin, and quercetin [16,20,21].

## 2. Phytochemical Constituents of *P. granatum*

Various parts, such as the fruit (arils & seeds), peel, flowers, and bark, of *P. granatum* contain different phytochemicals. The fruit consists of anthocyanins, polyphenols, polysaccharides, ascorbate, pectins, vitamins, organic acids, fatty acids, and malate [8,22,23]. The juice (part of the fruit) of *P. granatum* contains 85.4% water, approximately 1% polyphenols, 10.6% sugars, and 1.4% pectin. The juice is rich in minerals and contains varying concentrations of elements such as cobalt, sodium, calcium, magnesium, cesium, selenium, and zinc [8]. The seeds also possess an antioxidant capacity and a nutritional composition, such as sugars, vitamins, polyunsaturated fatty acids, polysaccharides, minerals, and polyphenols [24]. Approximately 80% of the seed oil is composed of a trienoic fatty acid, called punicic acid, which is capable of antitumor action [25]. The peel contains seven carbonic anhydrase inhibitors: highly active punicalin, tellimagrandin, pedunculagin, granatin B, punicalagin, and gallagyldilactone [26]. The rind (part of the pericarp/peel) contains ellagitannins and polyphenolic flavanols [27]. The flower contains ursolic acid, gallic acid, and triterpenoids, while the bark has ellagitannins, tannins, and alkaloids [22,23,28]. Table 1 comprehensively lists the phytochemicals of each part of the *P. granatum* [26] plant. These phytochemicals are not isolated to just *P. granatum* but are found in numerous other plants [29]. Many studies have been conducted on medicinal plants to investigate their anticancer therapeutic potential and mechanism of action, as indicated in Table 2.

## 3. *P. granatum* in Traditional Medicine

The traditional systems of medicine, such as Ayurveda, Traditional Chinese Medicine, and Unani, have used *P. granatum* for multiple purposes. The Ayurveda system, invented around 800 BCE, has been used as a traditional means of alleviating certain disease conditions to improve health [59]. It is accomplished through dietary controls, physical fitness, surgery, the management of stress, and herbal drugs. The drug preparation used in the system is derived from minerals, plants, and animal sources [59]. Unani was introduced by Muslims to India around a thousand years ago. It identifies the emotional, mental, physical, and spiritual causes of disease and well-being. The treatment is directed at self-healing through addressing lifestyle factors, e.g., eating healthier and regular exercise, but advanced disease medicine (herbal formulation) is also advised [59]. In Chinese medicine, diagnosis is through assessing four points: observations, patient questionnaires about their hearing and sense of smell, and taking the patient’s pulse. The treatment entails diet, herbs, acupuncture, etc., which are used for dysentery, pulmonary complications, infections (microbial, helminth), bleeding, etc. [59,60,61]. It can also be used for colic, menorrhagia, colitis, oxyuriasis, headache, allergic dermatitis, diuretic, acne, leprosy, piles, burns, snakebite, diabetes, and oral diseases [26,30]. In Chinese medicine, the *P. granatum* peel was used for its hemostasis, deworming, and antidiarrheal effects [62]. In Ayurveda, the *P. granatum* root and bark are believed to possess anti-parasitic and anthelminthic properties, and are therefore used in treating dysentery, ulcers, and diarrhea [62]. In Unani, the *P. granatum* flower is used for asthenia, while the seed formulation treats whooping cough, indigestion, vomiting, and nausea [63,64].

## 4. Photodynamic Therapy

The discovery of healing by sunlight can be traced from ancient times in Greece, India, and Egypt, and is known as heliotherapy [65,66]. The evolution of heliotherapy, later renamed phototherapy by Rikli, started with sunlight and now utilizes ultraviolet (UV) radiation [65]. Photodynamic Therapy (PDT) is an alternative method for non-malignant and malignant treatments. It utilizes light, a photosensitizer (PS), and oxygen to treat disease states [66,67,68]. The PDT mode of action entails cellular, vascular, and systemic immune levels of function, which may occur almost concurrently [66,67]. The cellular mechanism entails the elimination of tumors through necrosis and apoptosis. The necrosis of malignant cells occurs when a high-intensity light is introduced and causes quick cell destruction, in addition to a local and systemic immune response. Apoptosis in malignant cells occurs when a low light is introduced, and the cells stop their functions and go through programmed cell death. The immune response is not activated in apoptosis since no hazardous compounds are released from dead cells. The disruption in the vasculature of malignant cells caused by applying suitable light will lead to necrosis and, as a moderate reaction, apoptosis [67]. PDT is responsible for the activation of the immune system when necrosis is induced in malignant cells [69].

In PDT, a PS is introduced, and light of the required wavelength and intensity is applied to activate it. The PS can use multiple pathways to reach tumor cells, such as low-density lipoprotein receptor binding, lipid binding, uptake via tyrosine kinase, diffusion, etc. The photochemical reactions (type I and type II) are pathways that PS can go through and result in apoptosis or necrosis [63,66]. Aside from oxygen and light, the PS is the most vital part of the PDT mechanism. Clinically, only a limited number of PS are being utilized because of their particular specificity in cell uptake and photochemistry [66]. Photofrin is one of the most used and approved PS. Active research is still undergoing to identify other PS of clinical importance, and novel properties that would mitigate the limitations of poor chemical purity and insufficient penetration of the PS [70].

PDT is an alternative form of cancer treatment, and can be combined with other treatment options [16]. One such combination of medicine includes the use of phytochemicals. A study by Thakur and colleagues combined the PS, zinc phthalocyanine, with quercetin to improve the cancer-killing effects [19]. A study that combined quercetin and PDT with an aluminum phthalocyanine tetrasulfonate PS on human larynx carcinoma cells resulted in cell cytotoxicity [16]. Combining ellagic acid and PDT treatment on leukemia cells showed an improved induction of the cell apoptosis, which thus suggests that phytocompounds help to improve the therapeutic efficacy of the PDT [17].

## 5. Mechanism of Action and Therapeutic Properties of *P. granatum*

**Figure 2:**
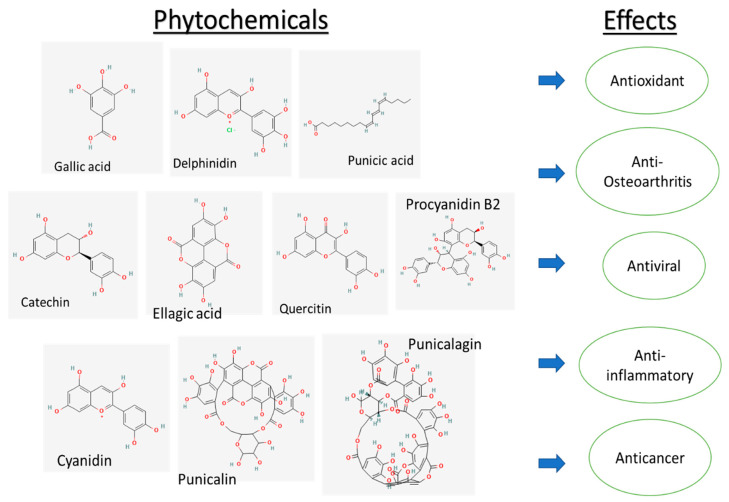
Phytochemicals found in *P. granatum* and its therapeutic properties.

Some of the phytochemicals of *P. granatum* can cause the down-regulation of extracellular, signal-regulated kinase ½ and c-Jun N-Terminal Protein Kinase 1, and up-regulation of tumor suppressor p53, which leads to cellular DNA damage [71]. These compounds induce therapeutic effects, such as antioxidant, anti-inflammatory, antiviral, anti-osteoarthritis, anticancer, etc., as shown below in Figure 2.

### 5.1. Anticancer Properties

**Figure 3:**
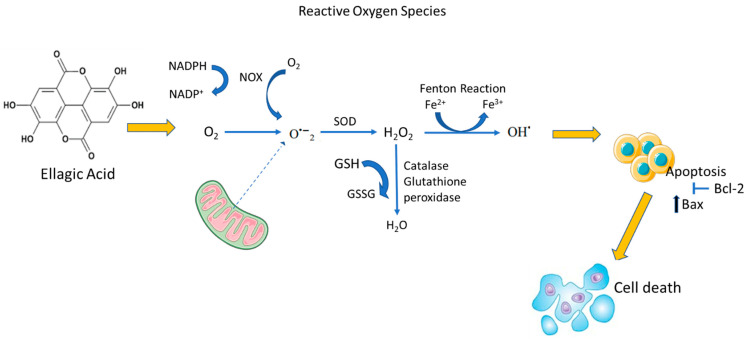
Mechanism of action of ellagic acid from *P. granatum* extract on MCF-7 breast cancer cells. Superoxide dismutase (SOD), oxidase enzyme (NOX), molecular oxygen (O_2_), glutathione (GSH), oxidized glutathione (GSSG), hydrogen peroxide (H_2_O_2_), superoxide anion (O_2_•−), hydroxyl ion (OH•), NADPH (nicotinamide adenine dinucleotide phosphate), nicotinamide adenine dinucleotide phosphate coenzyme (NADP^+^), ferrous cation (Fe^2+^), ferric cation (Fe^3+^), and water (H_2_O).

In a breast cancer study, the *P. granatum* pericarp phytochemical, genistein, was used to inhibit the proliferation of ER+ MCF-7 cancer cells. Genistein modulates the ER-α and ER-β selective estrogen receptors, and activates the cell cycle arrest and tumor suppression, respectively [8]. Another study that evaluated the antioxidant, antiproliferative, and apoptotic effects of the methanol extract from pomegranate peel found a decreased proliferative and increased apoptotic activities of MCF-7 human breast cancer cells [72]. These findings support the theory of the anticancer effect of *P. granatum*. The polyphenolic component, ellagic acid, in *P. granatum* also contributed to these observed impacts. These findings are further supported by works from Modaeinama and colleagues [73,74]. They reported that ellagic acid could induce the upregulation of Bax (Bcl-2-associated X) and Bcl-2 (B-cell lymphoma 2) proteins [73,74]. The expression of Bax (Bcl-2-associated X), a pro-apoptotic gene, was increased, while the anti-apoptotic gene, Bcl-2 (B-cell lymphoma 2), expression was decreased/inhibited, as depicted in Figure 3 [73].

The anticancer components of *P. granatum* are polyphenols, specifically ellagitannins (ET), flavonoids, punicalagin, to mention a few. The ET metabolize into active compounds named urolithin A (UA) and ellagic acid through the gut microbiota [9]. The urolithins suppress colon cancer cell proliferation, activate cell cycle cessation, and amend specific cellular processes linked with colon cancer progression, such as mitogen-activated protein kinase (MAPK) signaling [9]. A study using bioactive compounds of pomegranate, such as ellagitannins and punicalagin, showed that the inflammatory cell signaling in colon cancer cells was suppressed by significantly decreasing the cyclooxygenase-2 (COX-2) expression [75]. The ellagic acid metabolites of *P. granatum* ET have been shown to block intestinal inflammation, by suppressing the inflammatory mediators of inducible nitric oxide synthase (iNOS) and COX-2 [76].

In medicine, ellagic acid showed a probable chemo-preventive activity against prostate cancer. The inhibition of the motility and invasion of androgen-independent prostate cancer, PLS10 and PC3 cell lines, was seen when the cells were treated with a nontoxic concentration of ellagic acid. This was achieved by regulating the matrix metalloproteinases [74]. The study of the effect of pomegranate peel polyphenols on prostate cancer cells showed that the extract inhibited the proliferation of the cells and activated their apoptotic mechanism. After treatment with *P. granatum* juice extracts (punicic acid, luteolin and ellagic acid), the chemotactic proteins, which play a role in metastatic cancer (prostate, breast, renal, and colorectal), were in decline. This was accomplished by inhibiting the stromal cell-derived factor 1 alpha, and blocking the proteins that signal the C-X-C chemokine receptor type 4 (chemotactic proteins) [77]. The treatment of prostate cancer in vivo and in vitro with *P. granatum* peel extract showed the presence of apoptosis when viewed under fluorescence microscopy [78]. The treatment of lung cancer cells (A549) with *P. granatum* leaf extract resulted in the inhibition of cell proliferation, apoptosis induction, and the inhibition of cancer spread [79].

Punicic acid is a conjugated linolenic acid that contributes to the anticancer effects of *P. granatum* seed oil [80]. The performed studies showed cytotoxic effects on cancer cells. Its mechanism of action is not clearly understood, as other phytochemicals can be responsible for cancer cell breakdown, but it can be speculated to involve cytokine regulation, apoptosis activation, and malignant cell proliferation suppression [80]. As shown through the different studies elaborated, *P. granatum* phytochemicals show anticancer effects in other cancers with promising results through modulation, inhibition, and promotion of different proteins, hormones, and enzymes.

### 5.2. Antioxidant Properties

Equilibrium between the generation and removal of free radicals is imperative; hence, the term oxidative cellular stress results in imbalanced reactive oxygen species (ROS) production. ROS includes charged species (hydroxyl and superoxide radical) and uncharged species (hydrogen peroxide and singlet oxygen) [81]. Reactive atoms or molecules with unpaired electron/s in their external shell are termed free radicals. They are formed during the interactions of specific molecules with oxygen. Radicals are produced when a molecule receives or gains an electron [7]. ROS are reactive radical derivatives of oxygen, while reactive nitrogen species (RNS) are non-radical derivatives of nitrogen. Reactive oxygen and nitrogen species can either be endogenous or exogenous and can cause oxidative alteration of major cellular macromolecules like lipids, DNA, carbohydrates, and proteins [82].

Natural antioxidants are found in fruits and vegetables and have been of medical interest due to their prevention of oxidative damage by utilizing their -OH group to scavenge reactive radicals [83]. Grapes, berries, pomegranates, oranges, spinach, cabbage, etc. are among those fruits and veggies [83,84]. These antioxidants comprise flavonoids and phenolic compounds [10]. In *P. granatum* antioxidant properties are found in ellagic acid, hydrolyzable tannins, punicalagin, punicic acid, and anthocyanins [83]. Althunibat and colleagues performed a study on oxidative damage in experimental diabetic rats, which showed improved activity of antioxidant enzymes such as catalase, glutathione-S-transferase, glutathione reductase, superoxide dismutase, and glutathione peroxidase [85]. There is feasible suppression of tissue damage and inhibition of organ dysfunction caused by chronic hyperglycemia through the improvement of the activity of antioxidant enzymes by peel extract of *P. granatum*. The phenolic components of *P. granatum* peel extracts have been found to act as free radical scavengers thus, reducing the toxicity of ROS generated [85]. The anti-inflammatory action of punicic acid works by suppressing tissue necrosis factor α, which induced an increase in NADPH oxidase and hydroxyl radical scavenging action [25,86].

### 5.3. Anti-Osteoarthritis Properties

Osteoarthritis is a chronic musculoskeletal disorder that affects about 1.71 billion individuals worldwide [87]. Osteoarthritis disrupts the equilibrium between the production and breakdown of extracellular matrix components by chondrocytes. Osteoarthritis is considered the most common form of arthritis, and the causative agent of this osteoarthritis is still largely unknown [88]. The main treatment option for osteoarthritis is disease management, a known cure [89]. This is in the form of treating symptoms like inflammation, and slowing disease progression with therapies like acupuncture, physical therapy, and drugs [90,91].

Phytochemicals like anthocyanins, tannins, and punicalagin in *P. granatum* are effective in treating arthritis and can be used as an alternative treatment [91]. Studies show the improvement of the molecular pathway responsible for the development of osteoarthritis when treated with *P. granatum* [92,93,94]. Mahdavi and Javadivala demonstrated that treatment with *P. granatum* juice improved osteoarthritis. This was observed through better- functioning chondrocytes, leading to reduced damage to proteoglycans [94,95]. Similarly, Liu and colleagues (2021) reported a decrease in the progression of osteoarthritis in their study due to the protective effect of Punicalagin on chondrocytes [93].

The study by Choi and colleagues on anti-arthritic effects of *Achyranthis radix*, pomegranate, and *Eucommiae cortex* extracts on the primary cultured rat articular chondrocytes showed an inhibition of inflammatory response and associated extracellular matrix degradation and chondrocyte apoptosis [92].

### 5.4. Anti-Inflammatory Properties

Inflammation is a natural response by the immune system against substances that seem foreign or are harmful to the body, and is vital for tissue repair [27]. Acute and chronic inflammation are the two phases in the inflammation process. Innate immunity is an inflammation that occurs for a short duration and is advantageous to the host’s health. Chronic inflammation persists for longer, predisposing the host to various chronic illnesses, including cancer [27].

Okada, and Shimizu and colleagues have studied the relationships between cancer and inflammation, which suggested that elevated levels of inflammatory cytokines are responsible for cancer formation in low-grade chronic inflammation [96,97]. This accounts for an estimated 20–25% of cancer cases caused by a microbial infection inflammation [97]. The research has shown that the transcription factor, nuclear factor kappa B (NF-κB), the most recognized molecule, links the inflammation and cancer initiation, specifically tumor progression [96].

Houston and colleagues studied the anti-inflammatory action of *P. granatum* pericarp extract, and the results showed that the tannins (80% punicalagin and 1.3% ellagic acid) could cause the downregulation of COX-2 [27]. The punicic acids and their counterparts from pomegranate oil were used to treat cancer cell lines (breast, colon, prostate, and liver), decreasing the pro-inflammatory cytokines [36,37]. Ellagitannins and ellagic acid were utilized to treat intestinal colitis-induced inflammation and ulcers, with results showing the inhibition of HIF1α, which can be responsible for the colitis-induced inflammation, induction of tumor suppression, and decrease in the cytokines expression [98]. Osteoarthritis can advance due to damage to chondrocytes caused by inflammation in the disease. The treatment with ellagic acid caused the inhibition of NF-κB [99]. Ben-Saad and colleagues conducted a study that showed the suppression of cytokines and inflammatory mediators, such as nitrous oxide, using gallic acid, punicalagin, and ellagic acid found in *P. granatum* [100,101]. The literature on the anti-inflammatory action of *P. granatum* phytochemicals shows promising results, with possible future consideration for clinical trials after sufficient in vivo and in vitro studies.

### 5.5. Antiviral Properties

Research on *P. granatum* on viruses (herpes simplex virus, influenza virus, and human deficiency virus) was done by (Moradi et al., 2019; Howell and D’Souza, 2013) [27,102,103] and their findings showed a decrease in the viral titer load. The pomegranate peel inhibited replication against the influenza virus [103]. Evidence showed that punicalagin in *P. granatum* was an effective anti-influenza that blocked the virus’s RNA replication and inhibited red cell agglutination in chickens. The potential of effective viral treatment in human immunodeficiency virus (HIV) is postulated by Kotwal, Neurath, and colleagues due to the pomegranate’s potential to neutralize infectivity and block binding of HIV-1 to a cluster of differentiation 4 (CD4) receptors [102,104].

In the era where we find resistant strains of influenza, natural remedies can be explored as an alternative treatment. Flavonoids such as catechin, quercetin, rutin, and prodelphindin, found to have antioxidant, anti-inflammatory, antibacterial, antineoplastic, and antiviral properties, can be used for influenza treatment [105]. Influenza-infected MDCK cells treated with pomegranate peel extract (PPE) exhibited viral adsorption and RNA transcription inhibition [105]. Punicalagin was utilized against the alphavirus Mayaro virus, resulting in antiviral effects [106]. Phenolic components (*n*-butanol and gallic acid) caused inhibition of virus replication in adenovirus [107]. The antiviral effect of *P. granatum* is through the inhibition of replication and does not necessarily entail virucidal action [107].

### 5.6. Toxicity of P. granatum

The medical community has focused on herbal drugs as an alternative to synthetic pharmaceuticals for treating diseases. Natural remedies were employed in the past, and some traditional medicine systems are still being used today in countries, such as India and throughout Asia, benefiting human health. For patient safety, the toxicity of herbal therapies needs to be evaluated. The studies on the toxicity of *P. granatum* have been carried out by various authors and tabulated in the table below (Table 3).

## 6. Conclusions and Future Considerations

Photodynamic therapy has been of much interest to the medical community, due to its benefits in cancer treatment with minimal surgery requirements, reduced systemic toxicity, and its overall reduction in side effects. Improvements in its efficacy can lead to better survival statistics. Plants are the next alternative treatment option, due to their anticancer properties. Natural plants, such as *P. granatum,* contain phytochemicals such as flavonoids, phenolics, and ellagic acid, which are responsible for the cytotoxicity of malignant cells. The research has shown its role in cancer cell proliferation and apoptotic cell death pathway activation.

*P. granatum*’s ability as an antioxidant, antitumor, and anti-inflammatory agent has shown a cancer cell DNA fragmentation activity, reduction in tumor cell growth, an inhibition of NF-κB, and activation of ROS production. We have discussed this plant’s therapeutic properties, as reported in much of the research. Still, more is needed as the scientific community continues to explore the potential of *P. granatum* in combination therapies with photodynamic therapy to enhance its killing effect. The clinical studies geared toward treating with *P. granatum* have included preclinical and clinical trials of diseases such as inflammation, cancer, cardiovascular disorders, metabolic disorders, and infections, to name a few [113]. The current studies focus on the different parts of the pomegranate plant, including the peel [73,114,115], juice [75,93,116], leaf [79], etc.

Other studies on several phytochemicals for their beneficial properties are necessary to eliminate the toxicity of chemically synthesized drugs, especially the ones used for cancer treatment. *P. granatum* phytotherapy can be combined with surgery, immunotherapy, and hormonal therapy to maximize its efficacy and achieve better patient survival. The effective dose for treatment is an important aspect that needs to be explored in using *P. granatum* for cancer treatment. If all these areas are factored in the future, *P. granatum* will be a better plant source for alternative cancer treatment.

## Figures and Tables

**Table 1:** Phytochemical components of *P. granatum*.

Plant Parts	Phytochemicals	Reference
**Bark**	Ellagitannins and Gallotannins: brevifolin, castalagin, carboxylic acid, punicalagin, galloylpunicalin castalagin; Alkaloids: serine, hygrine, pseudopelletierine; Sterols and Terpenoids: friedooleanan-3-one	[30,31]
**Peel**	Catechin: gallocatechin; Ellagitannins and Gallotannins: granatin b, pedunculagin, punicalagin; Flavonoids: delphinidin, pelargonidin, quercetin; Tannins: Phenolic acid: ellagic, chlorogenic	[32,33]
**Fruit**	Ellagic acid derivatives: ellagic acid; Ellagitannins and Gallotannins: corilagin; Flavonols: kaempferol, quercime, ritrin	[2,34]
**Seed**	Ellagic acid derivatives: ellagic acid; Fatty Acids and Triglycerides: conjugated linolenic acid, tri-O-punicylglycerol, palmitic acid; Sterols and Terpenoids: estrone, testosterone	[35,24,26,36,37]
**Juice**	Catechin and Procyanidins: catechin, procyanidin B1 and B2; Anthocyanins and Anthocyanidins: anthocyanins, cyanidin, delphinidin; Organic Acids: chlorogenic acid, citric acid, gallic acid; Flavonoid: quercetin, rutin	[26,38]
**Root**	Alkaloids: norhygrine, isopelletierine, hygrine, pelletierine	[39,40]
**Leaves**	Ellagitannins and Gallotannins: punicalin, tellimagrandin, punicafolin, tercatain; Flavonols: apigenin-4′-o-β-d-glucoside, luteolin-3′-o-β-d-glucoside; Simple Gallyol Derivatives: brevifolin	[41,42]

**Table 2:** Exemplary the Medicinal Plants and Its Bioactive Compounds.

Plant Name	Bioactive Compounds	Mechanism of Action	Cancer Types	Reference
*Beta vulgaris* L.	Betaine, *p*-coumaric acid, rutin, kaempferol, rhamnocitrin, syringic acid, astragalin, oleanolic acid, β-carotene, caffeic acid, lutein, rhamnetin, betalains, ferulic acid	Cytotoxicity activity is caused by methylation of DNA in cancer cells. These compounds also showed scavenging activities of free radicals, inhibition of NF-κB, and DNA intercalation.	Skin and lung cancer	[43,44,45]
*Allium sativum* L.	Organosulfur, polysaccharides, saponins and phenolic compounds	Blockage of G_2_/M phase of cell cycle and inhibition in tumor growth.	Bone cancer	[46,47,48]
*Annona muricata* L.	Alkaloids, phenols, kauranes, flavonoids, lignans, megatigmanes, terpenoids, acetogenin, tannins, glycosides, cyclopeptides, and oils	Reduced mitochondrial membrane integrity and ATP production and induction of apoptosis.	Breast cancer	[49,50]
*Daucus carota* L.	Phenols, ascorbic acid (vitamin C), carotenoids, and polyacetylenes	Blocking of cell proliferation by apoptosis and cell cycle cessation of cancer cells.	Colorectal cancer	[51,52]
*Artemisia annual* L.	Arteannuin B, scopoletin, artemisinin and arteannic acid	Cell viability inhibition, activation of caspase 3 and fragmentation of DNA leads to apoptosis.	Prostate, lung, and breast cancer	[53,54]
*Kigelia Africana*	Terpenoids, steroids, flavonoids, phenols, furanonaphthoquinoids, coumarins, fatty acids, caffeic acid norviburtinal, and iridoids	Inhibition of cell viability and proliferation.	Skin and renal carcinoma	[55,56]
*Opuntia* spp.	Ascorbate, flavonoids, carotenoids, phenolic acids, kaempferol, and betalains	DNA fragmentation and cell arrest at the G_2_/M phase	Prostrate and breast cancer	[57,58]

**Table 3:** Toxicity studies of *P. granatum*.

Research Topics	Results Found	Reference
Acute and subacute toxicity study of the ethanol extracts of *P. granatum* (Linn) whole fruit and seeds and synthetic ellagic acid in Swiss albino mice	No adverse effects were found, and it was classified as non-toxic, and safe to utilize. The dosage used was 2000 mg/kg of body weight of the extracts.	[108]
Evaluation of the antidiabetic, hypolipidemic, and antioxidant activity of hydroalcoholic extract of leaves and fruit peel of *P. granatum* in male Wistar albino rats	No toxicity effects were found. The highest dose administered was 2000 mg/kg body weight of the extracts.	[109]
*P. granatum* peel extract toxicity in mice	No adverse effects were found on the utilization of *P. granatum* in mice with a maximum oral dose of 7.5 mg/kg or intravenous amount of 224 mg/kg body weight.	[110]
Evaluation of antibacterial activity and acute toxicity of pomegranate (*P. granatum* L.) seed ethanolic extracts in Swiss webster mice	The toxicity test showed a positive result. Results showed that only a high systemic dose would cause death, LD_50_ was assumed to be greater than 2000 mg.	[111]
Toxicological assessments of a proprietary blend of *P. granatum* fruit rind and *Theobroma cacao* seed extracts: acute, subchronic, and genetic toxicity studies	No toxicity was found during testing at 5000 mg/kg body weight of the extract.	[112]

## Data Availability

Not Applicable.

## List of Abbreviations

ER+	Estrogen receptor-positive
Erα	Estrogen receptors alpha
Erβ	Estrogen receptors beta
Bcl-2	B-cell lymphoma 2
Bax	Bcl-2-associated X
ET	Ellagitannins
UA	Urolithin A
MAPK	Mitogen-activated protein kinase
ERK	Extracellular signal-regulated kinase
COX-2	Cyclooxygenase-2
iNOS	Inducible nitric oxide synthase
MCF-7	Breast cancer cell line
PC3	Human prostate cancer cell lines
HIF1α	Hypoxia-inducible factor 1-alpha
ROS	Reactive oxygen species
RNS	Reactive nitrogen species
NF-κB_	Nuclear factor kappa B
IL-1β_	Interleukin-1β
HIV-1	Human immunodeficiency virus
CD4	Cluster of differentiation 4
MDCK	Madin-Darby canine kidney
PPE	Pomegranate peel extract
